# Plaidoyer pour une intégration de l'albinisme oculocutané au sein des Maladies tropicales négligées dermatologiques

**DOI:** 10.48327/mtsi.v3i4.2023.434

**Published:** 2023-10-24

**Authors:** Robert AQUARON, Patricia LUND, Charlotte BAKER

**Affiliations:** 1 Faculté de médecine, Aix-Marseille Université, Marseille, France; 2 Department of Biomolecular and Sports Science, Coventry University, Royaume-Uni; 3 Department of Languages and Cultures, Lancaster University, Royaume-Uni

**Keywords:** Albinisme oculocutané, Cancers cutanés, Morbidité, Mortalité, Mysticisme, Meurtres rituels, Associations de patients, Maladies Tropicales Négligées, Afrique subsaharienne, Oculocutaneous albinism, Skin cancers, Morbidity, Mortality, Mysticism, Ritual murders, Persons with albinism associations, Neglected Tropical Diseases, Sub-Saharan Africa

## Abstract

L'albinisme oculocutané (AOC) est une affection génétique qui, spécialement en Afrique subsaharienne, devrait être considérée comme une Maladie tropicale négligée (MTN). Le développement de cancers cutanés est la complication majeure chez les sujets albinos, aboutissant très souvent à leur décès. Cette létalité est bien connue et a été signalée dans de nombreuses publications médicales.

Dans ces pays, les personnes albinos font très souvent l'objet de discriminations culturelles, sociales, médicales, morales et économiques. La personne albinos est considérée comme un « Africain blanc », une créature intermédiaire entre les humains et les esprits/génies, dotée de pouvoirs innés pouvant faire le bien et le mal. Cette particularité a fait du corps de l'individu albinos l'objet de mutilations, de violences sexuelles et/ou de crimes rituels en vue d'utiliser certains organes pour la préparation de talismans supposés porter chance, santé et prospérité.

Sous l'influence d'ONG internationales et d'associations africaines de personnes albinos, francophones, anglophones et lusophones, les instances de l'ONU, l'OMS (Organisation mondiale de la Santé) et l'UNESCO (United Nations Educational, Scientific and Cultural Organization) ont réagi. Le 13 juin 2013, une résolution pour combattre ces agressions et discriminations a été votée. Dès lors, cette date est devenue la « Journée internationale de sensibilisation à l'albinisme ». Elle est fêtée avec éclat et panache, en particulier par les nombreuses associations des pays d'Afrique subsaharienne.

L'OMS vient d'ouvrir en juin 2022 un cadre stratégique pour le contrôle et la gestion des MTN se manifestant principalement au niveau de la peau. Bien que ce projet soit limité actuellement aux dermatoses de nature infectieuse, nous développons dans cette tribune notre plaidoyer pour l'intégration de l'AOC parmi les MTN.

## Abstract (en)

Oculocutaneous albinism (OCA) is genetically transmitted. In this paper we advocate for this disease to be included in the NTD list of the WHO. OCA type 2 is the most common form of albinism in sub-Saharan Africa, with a prevalence of 1 in 7900 among the Bamileke of Cameroon, 1 in 3900 in South Africa and 1 in 1100 among the Ibos of Nigeria, as compared to a prevalence of 1 in 10,000 among African Americans and 1 in 36,000 among White Americans and Europeans. The medical problems related to ophthalmological aspects (poor visual acuity, ametropia, nystagmus, photophobia) and dermatological aspects of albinism (sensitivity to UV rays from the sun and development of skin cancers) are well known. However, their management is often challenging for persons with albinism in sub-Saharan Africa because of their financial burden and the difficulty of accessing medical specialists. In many African countries, persons with albinism are also very often the subject of social, cultural, medical, moral and economic discrimination, which can limit their access to education, employment and community life. They are considered ‘white Africans’, intermediary and incomplete, with innate powers for good and evil. This particularity has made persons with albinism the targets of mutilations and/or ritual attacks for the purposes of using their body parts in the preparation of charms to bring good luck, health or prosperity. On 13 June 2013, as a result of lobbying by the Canadian NGO Under the Same Sun and African albinism associations, United Nations bodies including UNESCO and the WHO (World Health Organization) responded and a Resolution addressing the discrimination and attacks was voted in. The date has since become International Albinism Awareness Day and is celebrated on a different theme each year with great energy and impact, especially by French, English and Portuguese speaking albinism associations across sub-Saharan Africa. In 2015 the Human Rights Council created the position of Independent Expert on Albinism to better collect and analyse data on the rights of persons with albinism around the world, and especially in countries where ritual attacks occur. The data collected by albinism associations and the authorities thus go directly to the UN Human Rights Directorate. Despite this international attention to the attacks on persons with albinism, one of the biggest threats is skin cancer, which very often leads to early death. In 2022, the WHO launched a strategic framework for the control and management of neglected skin-related neglected tropical diseases – an additional reason to include oculocutaneous albinism as an NTD. Although the focus is currently limited to dermatoses of an infectious nature, we argue here for the integration of oculocutaneous albinism among NTDs because the deadliness of these carcinomas in sub-Saharan Africa is well-known and has been examined in a number of medical publications. Here, we propose that oculocutaneous albinism in sub-Saharan Africa be classified as an NTD to help people with albinism have access to health, economic, social and cultural rights.

## Introduction et historique

L'histoire de l'albinisme remonte « à la nuit des temps » [51] puisque la première personne albinos signalée dans un écrit fut Noé, d'après la description qu'en donne le livre d'Hénoch, qui était son arrière-grand-père: « Quand l'enfant naquit, son corps était plus blanc que neige et plus rouge qu'une rose, toute sa chevelure était blanche comme de blancs flocons, bouclée et splendide. Et quand il ouvrit les yeux, la maison brilla comme le soleil » [51]. C’était déjà une bonne description clinique de l'albinisme.

En 1572 les navigateurs portugais débarquant sur les côtes du royaume de Loango, situé actuellement entre la République congolaise (RC) et la République démocratique du Congo (RDC), observèrent des Africains blancs. C'est le père jésuite Balthasar Telles qui leur donna le nom d'albinos, du latin *albus,* blanc [47]. C'est avec le développement des salons pendant le siècle des Lumières que le phénomène albinos africain gagne l'Europe et en particulier la France [2, 7]. L'espace mi-public, mi-privé des salons réunit des personnalités de tous horizons: acteurs, savants, écrivains, artistes, penseurs, polémistes qui débattent de sujets divers propres à leur profession. C'est ainsi que Voltaire écrit en 1734 dans ses *Lettres philosophiques*: « On m'assure que la race de ces petits maures blancs est fort fière, qu'elle se croit privilégiée du ciel ». Dans son livre *La Vénus physique,* Maupertuis tente en 1745 d'expliquer la diversité des races humaines et la couleur de la peau des premiers êtres humains ainsi que le phénomène albinos: « J'aimerais bien mieux m'occuper du réveil d'Iris que de parler du petit monstre dont il faut que je vous conte l'histoire. Cet enfant de 4 ou 5 ans, qui a tous les traits d'un nègre, possède une peau très blanche. Il fut apporté à Paris en 1744. Sa tête est couverte de laine blanche tirant sur le roux. Il est né d'une mère et d'un père africains très noirs ». D'Alembert et Diderot écrivent dans leur encyclopédie (1751-1772): « On soupçonne les albinos d’être des animaux métissés issus d'une femme et d'un orang-outan. » En 1766, Linné fait des personnes albinos une espèce particulière du genre humain. Il leur donne le nom *d'Homo nocturnus,* car ils se cachent le jour et sortent la nuit pour chercher leur nourriture. Cette appellation n'est pas sans rappeler les Indiens Cuna des îles San Blas au Panama, qui sont appelés « les enfants de la lune » car ils vivent à l'intérieur de leur habitation pendant la journée pour se protéger du soleil et sortent la nuit où ils voient plus clair, spécialement lorsque la lune est là [27, 28]. Il faudra attendre 1879 pour que Broca, médecin très connu pour sa découverte dans le cerveau humain du centre de la parole dit « aire de Broca », inaugure la période clinique [13]. Il était également un anthropologue renommé qui a précisé les recherches anthropologiques cliniques à réaliser chez les sujets albinos: « L'albinisme, général ou partiel, est-il rare ou commun ? Que donnent les unions contractées entre deux individus albinos ? Les albinos qui s'unissent à des individus non albinos transmettent-ils quelquefois leur anomalie à leurs enfants ? Les albinos paraissent-ils inférieurs aux autres individus de même race: vitalité, intelligence, fécondité, longévité ? L’état de la vision devra être étudié au grand jour, puis au demi-jour et enfin à l'obscurité ». Enfin c'est Gabriel Bertrand, chef du laboratoire de chimie biologique de l'Institut Pasteur à Paris qui découvre en 1896 la tyrosinase, une enzyme qui catalyse la formation du pigment noir, la mélanine [9]. C'est le début de la période biologique. L’étude de l'albinisme humain et sa répartition dans le monde jusqu'en 1911 sont très bien décrites dans un livre qui compte 6 tomes [46]. L'albinisme est aussi appelé albinisme oculocutané (AOC) pour bien préciser les deux principaux organes atteints, la peau et les yeux. Il a fallu attendre les années 1960 pour que l’étude des sujets albinos vivant en Afrique subsaharienne prenne à la fois un aspect médical (clinique et génétique) et psycho-social en Afrique du Sud. Ce pays disposait d'un institut de génétique très performant à l'Université du Witwatersrand à Johannesburg, dirigé par le Pr. T. Jenkins et comportant plusieurs chercheurs spécialisés dans le domaine de l'albinisme: J. Kromberg, P. Manga, R. Kerr, E. Zwane, M. Ramsay. De nombreux travaux ont été publiés [8, 20, 33, 37, 52] et ont été rassemblés dans un livre édité récemment [32]. Ces études se sont ensuite étendues aux pays anglophones voisins comme le Zimbabwe [14, 36, 48], le Malawi, la Zambie, la Tanzanie, puis aux pays francophones de l'Afrique de l'Est et du Centre – Cameroun [2, 6, 15, 48], Gabon, République centrafricaine, République du Congo, République démocratique du Congo – et de l'Afrique de l'Ouest – Nigéria [19, 43], Togo, Bénin, Côte d'Ivoire, Mali, Burkina Faso, Sénégal – sans oublier les pays lusophones, Angola et Mozambique. L'AOC de type 2 (AOC2), que nous définirons plus tard, est la forme la plus fréquente en Afrique subsaharienne, environ 98% en raison de la consanguinité fréquente lors des unions intra-ethniques: elle touche 1 personne sur 7900 chez les Bamilékés du Cameroun, 1/4182 au Zimbabwe, 1/3900 en Afrique du Sud, 1/3219 en Tanzanie et 1/1100 chez les Ibos du Nigéria. Signalons que la fréquence est de 1/10 000 chez les Afro-américains alors qu'elle est de 1/36 000 chez les sujets d'origine européenne [48].

## Albinisme et maladies tropicales négligées

Le concept de maladie tropicale négligée (MTN) a été structuré autour d'un axe fondateur: l'impact de ces maladies sur les droits de l'homme. Paul Hunt, alors membre du Comité des Nations unies sur les droits économiques, sociaux et culturels et rapporteur spécial sur le droit au meilleur état de santé, a contribué à cette réflexion [25]. Il existe des liens évidents entre les maladies négligées et les droits de l'homme. Les maladies négligées sont plus susceptibles de se produire lorsque les droits de l'Homme tels que les droits à la santé, à l’éducation et au logement, ne sont pas garantis. En outre les maladies négligées entraînent souvent des violations des droits de l'homme et des libertés fondamentales, y compris l’égalité et la nondiscrimination. Un site de l'OHCHR (Office of the United Nations High Commissioner for Human Rights) est dédié aux personnes albinos: http://albinism.ohchr.org/fr.

Une liste non exhaustive de 20 maladies a été établie par l'OMS qui comprend principalement des parasitoses (leishmaniose, bilharziose, trypanosomiase) et des maladies infectieuses (trachomatose, dengue, ulcère de Buruli). Ce concept a été récemment élargi au domaine des pathologies non infectieuses comme les envenimations en 2019 et les maladies de peau en 2022 [44, 45]. Pour l'instant, le cadre stratégique se limite aux dermatoses tropicales négligées d'origine infectieuse, quelquefois déjà citées précédemment (ulcère de Buruli, lèpre, filariose, gale, pian). Une journée internationale dédiée aux MTN a été votée par la World Health Assembly (WHA) en mai 2021 et se tient tous les 30 janvier.

**Figure 1:**
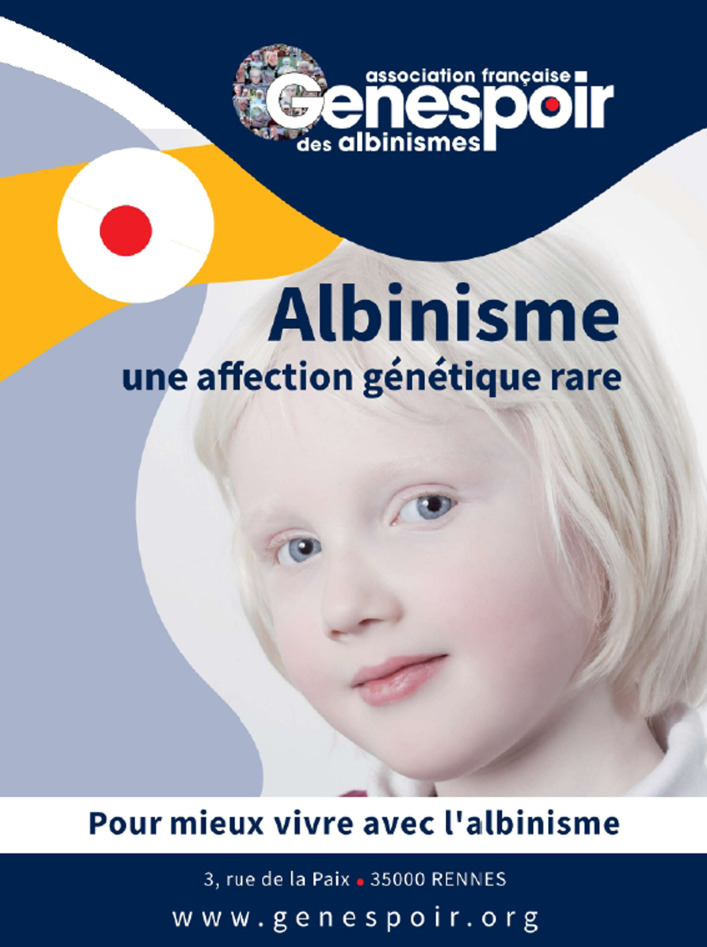
Page de couverture de la brochure publiée par Genespoir, l'Association française des albinismes Cover page of the brochure published by theFrench Albinism Association, Genespoir

En ce qui concerne l'albinisme oculocutané, le terme de « maladie » est souvent omis du fait de sa connotation négative et remplacé par « affection », « condition » ou « anomalie » sauf lorsque l'on le cite comme faisant partie des « maladies rares » et bientôt, nous le souhaitons, des « maladies tropicales négligées » (Fig. 1). En 1879, Broca écrivait déjà: « L'albinisme est toujours congénital. C'est une anomalie et non une maladie. [13] » Bien qu’étant une affection génétique de transmission autosomique récessive, nous proposons que l'albinisme oculocutané en Afrique subsaharienne soit classé dans le cadre des MTN, car les personnes albinos doivent avoir accès à tous les droits précédemment énoncés: économiques, sociaux, culturels (absence de discrimination) et relatifs à la santé (individuelle et collective).

Où en est-on à présent de l'albinisme oculocutané ? Si les problèmes médicaux liés aux aspects ophtalmologiques (vision faible, amétropie, nystagmus, photophobie) et dermatologiques (sensibilité aux rayons UV du soleil et développement de cancers cutanés) sont bien connus, leur prise en charge reste toujours délicate en raison de la difficulté d'accès aux spécialistes et de leur charge financière [1]. Ces faits ont conduit dans un premier temps à la formation d'associations privées d'aide aux personnes albinos pour les encadrer ainsi que leurs familles. Concomitamment, ces associations ont fait appel à divers ministères étatiques pouvant prendre en charge certains aspects de l'albinisme (santé et solidarité, éducation, social) pour finalement arriver aux instances internationales qui ont en charge les mêmes aspects de santé, d’éducation, de science et de culture comme l'OMS et l'UNESCO.

## Contrôle et prise en charge des anomalies dermatologiques

**Figure 2:**
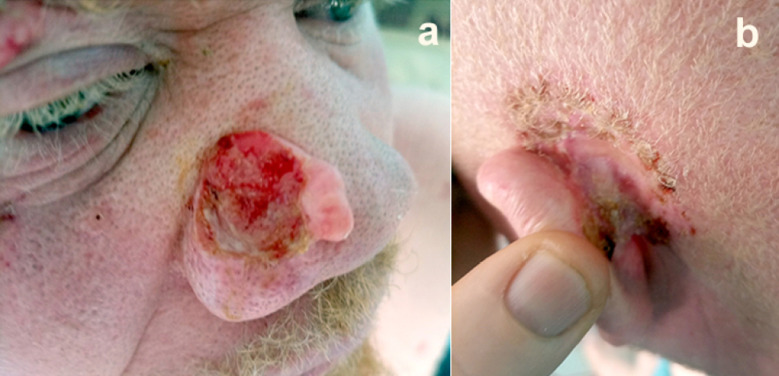
Cancers du nez (a) et de l'oreille (b) chez un sujet albinos Camerounais, d'ethnie Mbo et demeurant à Douala, quelques mois avant son décès en 2022 à l’âge de 40 ans (crédit photo: Claire Ngando) A Cameroonian person of Mbo ethnicity and living in Douala with albinism with cancers of the nose (a) and of the ear (b), a few months before his death in 2022 at the age of 40 (photo credit: Claire Ngando)

Les aspects dermatologiques des personnes albinos sont au premier plan de leur santé. L'absence de mélanine dans la peau rend celleci très sensible aux effets nocifs des rayons UVA (320-400 nm) et UVB (280-320 nm) du soleil, lesquels génèrent des anomalies dans l'ADN des noyaux des kératinocytes. La première étape se manifeste par le classique « coup de soleil » ou érythème solaire. Dans une deuxième étape, la peau réagit naturellement en s’épaississant par une prolifération des kératinocytes pour mieux se protéger. La peau se durcit. C'est la kératose actinique, une lésion pré-carcinomateuse. La troisième étape est l'atteinte des couches profondes de l’épiderme, *stratum spinosum* et *stratum basale,* et le développement de cancers cutanés ou carcinomes spinocellulaires et/ou basocellulaires. Ils sont la principale cause de décès des sujets albinos (Fig. 2).

Il est donc important d'informer les parents des enfants albinos et les personnes albinos de la dangerosité des expositions solaires dès la naissance. Il faut également les convaincre de l'efficacité des mesures de protection: ombrelles, chapeaux, vêtements couvrants, crèmes solaires. Les crèmes solaires photoprotectrices sont caractérisées par un pouvoir photoprotecteur supérieur à 50% (SPF 50+, Sun Protective Factor), mais sont coûteuses et hors de portée de la bourse des personnes albinos africaines. Aussi, avec l'aide d'associations comme l'ANACI (Association nationale des albinos de Côte d'Ivoire) à Abidjan, les Africains ont participé *in situ* à la préparation de crèmes photoprotectrices préparées à partir de corps gras locaux très réputés comme le beurre de karité. Celui-ci est extrait des noyaux des fruits d'un arbre, *Butyrospermum parkii,* très répandu dans la savane d'Afrique occidentale et centrale. Il est appelé « arbre sacré » ou « l'or des femmes » car son beurre est préparé exclusivement par les femmes et jouit de propriétés adoucissantes, émollientes et photoprotectrices. Il est utilisé non seulement par les personnes albinos, mais aussi par les femmes de peau noire [10]. Le seul inconvénient est qu'il ne peut pas être conservé car il rancit vite. L'un d'entre nous (RA) connaît un médecin albinos à Douala, Cameroun, qui a une clientèle importante de patients albinos à qui il prescrit comme photoprotecteur le beurre de karité que l'on trouve facilement sur les marchés à prix abordable. La même association prépare avec l'aide de la Fondation Pierre Fabre des crèmes solaires stables contenant de la vaseline et deux photoprotecteurs minéraux avec le même SPF 50+ dont le prix de revient est accessible. Le même produit est développé par des associations du Mali, du Togo, de Tanzanie, d'Ouganda et du Malawi.

Il importe également de réaliser des contrôles médicaux réguliers, annuels ou bisannuels, afin de traiter rapidement tout début de carcinome, en général par une chirurgie d'exérèse, puis par radiothérapie et/ou chimiothérapie. Malheureusement les personnes albinos ont souvent des retards et des difficultés d'accès aux soins car ceux-ci sont payants. Toutes ces circonstances expliquent leur décès fréquent suite à ces carcinomes. Il existe de très nombreuses publications sur les cancers de la peau en Afrique subsaharienne. Nous en avons cité sept à titre d'exemple, diffusées entre 1989 et 2021 en Afrique du Sud, en Tanzanie, en RDC, au Togo et au Cameroun [16, 26, 30, 34, 35, 49, 54]. La prise en compte de la létalité importante de ces carcinomes doit être un des arguments les plus importants pour justifier l'intégration de l'AOC parmi les MTN. On peut dire qu'en Afrique l'albinisme est un véritable problème de santé publique [23], une urgence médicale et sociale selon Murray Brilliant, l'un des spécialistes mondiaux de cette affection [12].

Il existe également dans ces pays quelques cas de personnes albinos possédant soit une peau brune [29], soit une peau rousse [31] ne développant pas de cancers cutanés en raison de la présence de pigments photoprotecteurs. Le lancement récent par l'OMS, le 8 juin 2022, d'un cadre stratégique pour le contrôle et la gestion des MTN [45] qui se manifestent principalement au niveau de la peau, vient à point pour inclure l'albinisme oculocutané dans ce projet.

## Anomalies ophtalmologiques

Elles peuvent être classées en caractéristiques sensorielles (hypopigmentation du fond d’œil, photophobie, hypoplasie de la fovéa, troubles de la réfraction, strabisme) et motrices (nystagmus). Toutes ces anomalies sont dues à l'absence de mélanine au cours du développement embryonnaire. Les troubles de la réfraction sont responsables de la « vision trouble » ou amétropie. L'amétropie la plus fréquemment retrouvée est l'astigmatisme. L'acuité visuelle est très diminuée d'environ 50%. Par contre la vision des couleurs est en général normale [11, 24, 50]. Le dépistage des amétropies et leur correction sont nécessaires et primordiaux pour la bonne scolarisation des enfants. Le port de lunettes de soleil est également efficace pour diminuer la photophobie.

## Génétique médicale et moléculaire

L'albinisme oculocutané est une affection de transmission autosomique récessive, c'est-àdire que les deux parents sont obligatoirement des sujets hétérozygotes « Na » ou porteurs sains, autrement dit possédant un exemplaire du gène normal « N » dominant et un exemplaire du gène albinos « a » récessif. Chez les sujets africains, les parents porteurs sains ont une peau noire et rien ne les distingue des « parents normaux » NN. Suivant la loterie mendélienne, à chaque naissance il y a une chance sur quatre pour obtenir un sujet albinos « aa ». Lorsque dans une famille naît un enfant albinos, les parents reçoivent le conseil génétique suivant: à la prochaine grossesse vous avez 1 chance sur 4 de donner naissance à un enfant albinos. Cet avertissement n'est pratiquement jamais pris en compte dans les familles africaines, car la fécondité est un signe de bonne santé. Malgré les difficultés physiques et morales à surmonter pour élever un enfant albinos, les familles acceptent cette probabilité. Pour éviter cet écueil, une solution simple rencontrée par l'un d'entre nous au Cameroun (RA), c'est la répudiation de la femme par le mari.

Initialement, l'AOC était classé d'après l'aspect phénotypique, c'est-à-dire les caractères visibles – coloration de la peau, des cheveux, de l'iris – mais aussi par des tests chimiques simples comme la mise en évidence de l'activité de la tyrosinase dans les bulbes de cheveux [53]. Ce test a permis de classer les personnes albinos en 2 catégories: ceux donnant une réaction positive, dits Tyr-pos, et ceux donnant une réaction négative, dits Tyr-neg [53]. Après la mise en évidence de la localisation du gène de la tyrosinase sur le chromosome 11q14, de sa structure en 1988, puis la description du 1^er^ cas d'albinisme identifié à une mutation (on dit à présent « variant pathogène ») du gène de la tyrosinase en 1989, la communauté scientifique a adopté une classification moléculaire en fonction de la nature de la protéine mutée: l'AOC de type 1 est dû à l'inactivité de la tyrosinase, l'enzyme qui catalyse la 1^re^ étape de la mélanogenèse; l'AOC de type 2 résulte de l'absence d'activité de la protéine « P » qui régule la mélanogenèse [41]. L'AOC2 est le type d'albinisme le plus fréquent en Afrique subsaharienne (environ 98%). La mutation la plus fréquente (de 60% à 90%) en Afrique de l'Est, du Centre et du Sud, est une délétion de 2,7kb (kilobase). La particularité de cette mutation est sa spécificité parmi les populations bantoues et son absence en Afrique de l'Ouest [3, 18]. Cette délétion peut exister à l’état homozygote ou hétérozygote. Dans ce dernier cas la 2^e^ mutation responsable de l'AOC2, à présent qualifiée de « variant pathologique » peut être soit « faux-sens » (remplacement d'un acide aminé par un autre acide aminé dans la protéine « AOC2 » qui devient inactive) soit « non-sens » (remplacement du codon d'un acide aminé par un codon stop qui arrête la synthèse de la protéine « AOC2 » et l'inactive). Il est indiqué par un « X ». À titre d'exemple, voici deux variants pathologiques différents décrits chez deux sujets camerounais en utilisant la nomenclature à 3 lettres pour les acides aminés et un chiffre pour sa position: p. Thr404Met pour le sujet KF [21] et p. Arg165X pour le sujet BT [4]. Il existe actuellement 17 gènes impliqués dans la mélanogenèse dont le déficit conduit à différents types d'albinisme [40].

## Mythes relatifs à la naissance d'un enfant albinos en Afrique subsaharienne

La naissance d'un enfant albinos, fille ou garçon, de deux parents de peau noire est un évènement extraordinaire qui frappe les esprits [5]. L'enfant albinos possède tous les caractères morphologiques des parents mis à part la coloration blanche de la peau, blonde des cheveux et bleue ou noisette des iris. Pour essayer d'expliquer cette naissance inhabituelle, les Africains ont recours à diverses croyances ou mythes qui se situent avant et/ou pendant la grossesse et qui touchent principalement la mère: se moquer d'une personne albinos, avoir des relations sexuelles adultérines ou avec un homme blanc ou pendant la période des menstrues [7], avoir des rapports sexuels avec un *tokolosh,* esprit malveillant chez les Shona du Zimbabwe [8], ou un génie comme les *mamiwata* [42]. Le terme *mamiwata* vient du pidgin *mami water,* mère des eaux, utilisé pour désigner l'ensemble des génies des eaux. La naissance d'un enfant albinos est considérée soit comme un bonheur chez les Bandas de République centrafricaine, soit comme une malédiction qui peut aller jusqu’à l'infanticide chez les Haoussas au Cameroun [2]. Au Mali, c'est considéré comme « le fruit d'une souillure » ou simplement de la violation de graves interdits.

Considéré comme un « Africain blanc », la personne albinos n'en reste pas moins ressentie comme appartenant au monde magique, ce qui fait d'elle une créature humaine intermédiaire et inachevée, mi-homme, mi-esprit, dotée de pouvoirs innés, qui peut faire le bien ou le mal. Elle est affublée, par exemple au Cameroun, de termes jugés positifs comme « le Blanc », « le bon Blanc », ou plutôt négatifs comme « *nguengerou* » dans la langue locale ou « cochon gratté » en raison des démangeaisons fréquentes de la peau blanche [17]. Elle occupe une place intermédiaire entre les nains réputés stériles, et les jumeaux, produits d'une nature trop généreuse [2, 5]. On peut signaler à ce propos que des jumeaux albinos et uni-albinos (un noir, un albinos) ont été décrits chez les Bamilékés du Cameroun [6].

## Mythes sur les pouvoirs rituels des personnes albinos

Cette dualité d’êtres entre humains et esprits (ou génies), cette ambivalence entre maléfique et bénéfique ont fait des personnes albinos dès la naissance la proie de crimes rituels, de viols et de mutilations en vue d'utiliser certaines parties de leurs corps et de leurs organes (bras, jambes, organes génitaux) pour la préparation de talismans supposés porter chance, santé, prospérité, conjurer les maladies ou les mauvais sorts, sans compter les violences sexuelles. C'est ainsi qu'au Mali lors des périodes d'examen ou d’évènements politiques importants comme certaines élections, présidence de la République ou autres, les personnes albinos restaient cachées chez elles de peur d’être attaquées. Au Mali également, les pommades à base de graisse d'individus albinos étaient très appréciées (communication personnelle, RA). En Tanzanie près de Mwanza situé au bord du lac Victoria, région pauvre, le seul espoir de subsister réside dans le commerce du poisson et la recherche de diamants dans les mines. Pour ces deux raisons, les crimes rituels et les mutilations des personnes albinos sont malheureusement toujours pratiqués [5, 22]. Toutes ces exactions, jusqu’à la fin du xx^e^ siècle, étaient seulement connues des spécialistes (médecins, anthropologues) et diffusées dans les médias étatiques locaux, mais jamais au niveau international.

## Diffusion des exactions subies par les personnes albinos au niveau mondial

En 2008, des crimes rituels à grande échelle dans le nord-ouest de la Tanzanie et au Burundi, dans la région des Grands Lacs – Victoria et Tanganyika – ont été rapportés par Vicky Ntetema, une Tanzanienne responsable du bureau de la BBC à Dar-es-Salaam et par le journaliste tanzanien Richard Mgamba [22]. Dès cette période, des volontaires de la Croix-Rouge ont aidé matériellement les enfants albinos réfugiés dans une école locale pour handicapés près de Kigoma en Tanzanie. Salif Keita et Belete Geleta ont très bien rapporté le sort tragique des personnes albinos dans la région des Grands Lacs et le rôle joué par la Fédération internationale des Sociétés de la Croix-Rouge et du Croissant-Rouge [22]. Par la suite, le relais a été pris par une ONG canadienne opérant en Tanzanie, Under The Same Sun (UTSS) et par les associations de personnes albinos de la Tanzanie et du Burundi. Le président de l'UTSS, Peter Ash, albinos lui-même, a porté ce tragique évènement à la connaissance du président de la République de Tanzanie, M. Jayata Kikwele, qui a condamné vigoureusement ces assassinats et le commerce des parties de leur corps. Il a aussitôt proposé une personne albinos, Madame Al-Shymaa Kway-Geer, comme députée au parlement tanzanien. Ce fut un bel exemple de la reconnaissance de la « personne albinos ». Peter Ash a continué son action en faveur des personnes albinos en sollicitant les instances internationales, l'ONU, en particulier l'OMS et l'UNESCO. Son action a été déterminante pour l'adoption par l'Assemblée générale des Nations unies, le 13 juin 2013, d'une résolution au sujet des agressions et des discriminations à l'encontre des personnes albinos. Ces crimes rituels ont été décrits par l'UTSS dans 27 pays d'Afrique subsaharienne. Les associations africaines de personnes albinos ont également participé à la diffusion de ces exactions parmi lesquelles on peut citer: Albinos sans frontières (ASF) au Burundi, Association pour la promotion des albinos au Cameroun (APAC), Association of Persons with Albinism of Malawi (APAM). Dans les pays voisins de la Tanzanie, le Burundi et l'Est de la République démocratique du Congo, une quarantaine de personnes albinos, y compris des enfants, ont été atrocement mutilés. Il a également été rapporté, dans un article de *Jeune Afrique* d'octobre 2008 intitulé « Le soleil les tue, les hommes aussi », qu'au Burkina Faso certains convoitent la puissance sexuelle que sont censés recéler leurs organes génitaux.

**Figure 3:**
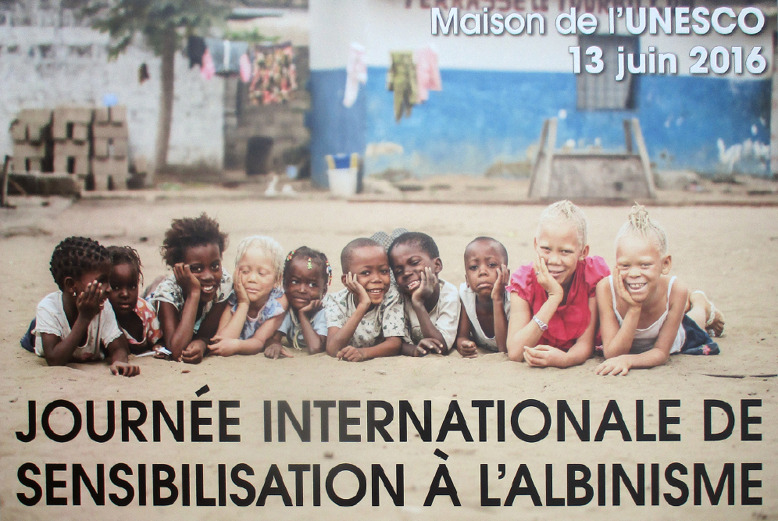
Affiche de la « Journée internationale de sensibilisation à l'albinisme » au siège de l'UNESCO, Paris, 13 juin 2016 (crédit photo: Patricia Willocq) Poster for ‘International Albinism Awareness Day’ at UNESCO headquarters, Paris, 13 June 2016 (photo credit: Patricia Willocq)

En 2014, l'Assemblée générale des Nations unies a proclamé le 13 juin « Journée internationale de sensibilisation à l'albinisme ». Cette date a été fêtée avec brio 2 ans plus tard au siège de l'UNESCO à Paris, le 13 juin 2016 (Fig. 3). Les allocutions de S.E.M. Jacques Kabale, ambassadeur et président du groupe africain, de Peter Ash, président de l'association UTSS, et de Firmin Matoko, sous-directeur général pour l'Afrique de l'UNESCO, ont inauguré cette journée au cours de laquelle différentes interventions ont eu lieu, réparties en 4 sessions: a) le panel des scientifiques et des médecins a traité de « La médecine face à l'albinisme »; b) le film documentaire « Black man white skin » a retracé le séjour en Tanzanie de médecins et chirurgiens espagnols pour soigner les cancers de la peau si fréquents chez les personnes albinos; c) l'exposition photo « Blanc ébène » a présenté de beaux portraits mettant en valeur la beauté attachante des personnes albinos; et d) le panel des associations de personnes albinos « L'albinisme au quotidien – témoignages » a rassemblé des associations françaises (Genespoir), canadiennes (UTSS) et africaines – Solidarité pour l'insertion des albinos du Mali (SIAM), Albinos de HEMA Nayélé à Banfora (AHB) et Association nationale pour l'intégration des personnes albinos (ANIPA) du Burkina-Faso.

Toujours dans ce cadre international, en 2015 le Conseil des droits de l'homme a créé un poste d’« expert indépendant sur l'albinisme » pour mieux analyser et recueillir les données concernant les droits des personnes albinos dans le monde et spécialement dans les pays où l'on pratique les mutilations et les crimes rituels (www.ohchr.org/fr/special-procedures/ie-albinism). Les données recueillies par les associations de personnes albinos et les autorités administratives des pays remontent ainsi à la direction des droits de l'homme à l'ONU. C'est une personne albinos du Nigéria, Ikponwosa Ero, qui a été nommée. Sa mère lui ayant dit que c’était un cadeau de Dieu, elle a vécu une enfance et une adolescence heureuse et normale, mais plus difficile à l'extérieur en raison des barrières dues à sa couleur de peau. Elle s'est fait connaître en travaillant avec l'association UTSS en Tanzanie et en visitant les personnes albinos de divers pays dont le Brésil en 2020 [38]. Après deux mandats de 3 ans, elle a été remplacée en 2021 par Muluka-Anne Miti-Drummond, une Zambienne de peau noire, spécialiste de droit international. Son premier voyage s'est déroulé à Madagascar en septembre 2022.

## Le 13 juin, journée internationale de sensibilisation à l'albinisme

**Figure 4:**
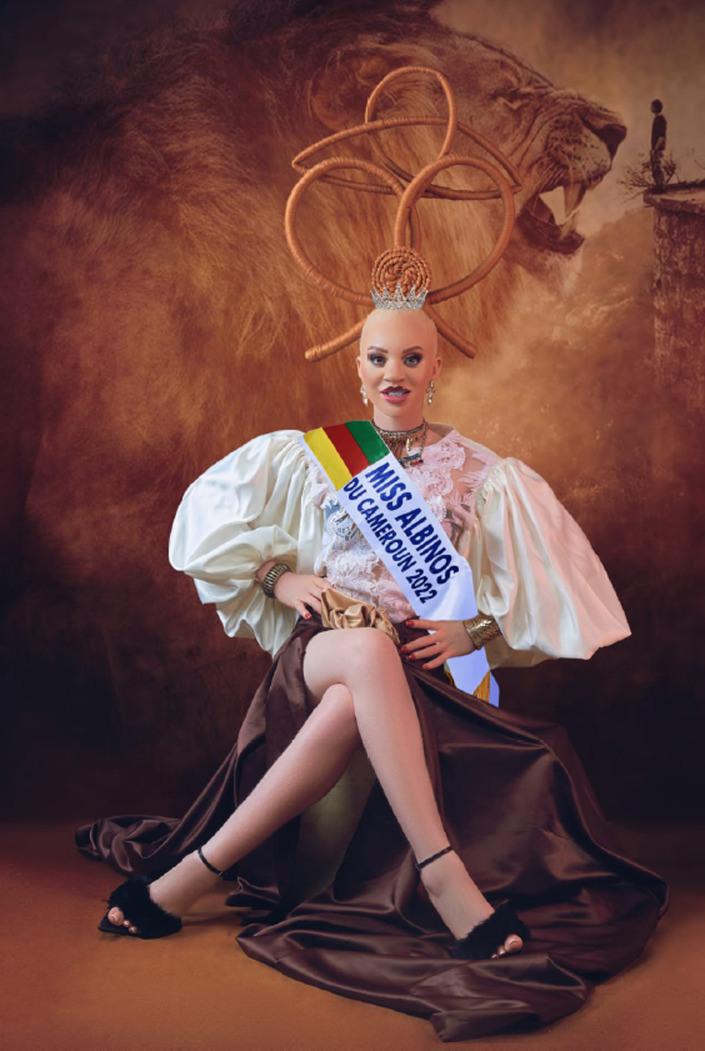
Miss Albinos du Cameroun, 2022 (crédit photo: Marie Madeleine Wafo) Miss Albinism Cameroon, 2022 (photo credit: Marie-Madeleine Wafo)

Le 13 juin est désormais célébré chaque année dans le monde entier par la communauté albinos, soit en présentiel, soit en distanciel. C'est une occasion pour se rencontrer et évoquer tous les problèmes médicaux et sociaux. Le thème de l'année 2022 était: « Unis pour faire entendre notre voix ». Cet évènement mondial, organisé par la GAA (Global Albinism Alliance) a pu être suivi *via* Facebook en quatre langues: français, anglais, espagnol et portugais. Il était présenté depuis Londres par le Dr Oscar Duke, une personne albinos britannique. Cette journée est aussi l'occasion dans certains pays comme le Cameroun d’élire une « Miss Albinos » pour mettre en valeur les femmes albinos (Fig. 4). Au Kenya l’élection comporte non seulement une « Miss albinos », mais aussi un « Monsieur albinos ».

## Les associations de personnes albinos

Il est important à ce stade-là de développer le rôle important qu'a joué la création de ces associations de personnes albinos dont les buts étaient: réunir les personnes albinos et leur famille, leur apporter les informations nécessaires sur la cause de leur albinisme, informer les parents sur la façon d’élever ces enfants en leur apprenant à se protéger du soleil pour éviter les futurs cancers de la peau en raison de l'absence du rôle protecteur de la mélanine. En leur indiquant également, avec l'aide des instituteurs puis des professeurs, de se placer au 1^er^ rang pour bien voir au tableau ou sur leur écran ainsi que de porter des lunettes de correction pour compenser leur faible acuité visuelle. Des lunettes de soleil teintées sont également recommandées afin d’éviter la photophobie. Il est important de signaler que plusieurs associations caritatives ont participé à des envois et/ou des apports sur place de ces deux types de lunettes aux personnes albinos. On peut citer Congo Action pour la RDC, Les écoles de l'espoir à Bukavu, RDC et à Bujumbura, Burundi, ainsi que Lunettes sans frontières. En Afrique subsaharienne, une association, dénommée Fondation Salif Keita pour les Albinos, a été créée à Bamako au Mali par ce célèbre chanteur albinos en 1993 afin de propager une image positive des personnes albinos: « Je suis noir, ma peau est blanche. » Vivant désormais en France, il a obtenu en 2009 un trophée aux Victoires de la musique pour son titre « La différence ». Actuellement, au Mali, au moins quatre associations sont actives chacune dans un domaine particulier ou sur une zone géographique différente: SIAM, AMPA, SOS Albinos, Fondation Salif Keita. En 1996 c'est à Yaoundé, Cameroun, que l'ASMODISA (Association mondiale pour la défense des intérêts et la solidarité des albinos) a vu le jour grâce à J. J. Ndoudoumou, albinos, conseiller auprès du Premier ministre. Par la suite, d'autres associations ont été créées par des personnes albinos tant à Yaoundé (ADESA, Association pour le développement et l’épanouissement social des albinos, Cameroun, APAC (Association pour la promotion des albinos camerounais, GICAS, Groupement d'initiative camerounaise des albinos et sympathisants) qu’à Douala (AFAD, Association des femmes albinos de Douala, Albi Care gérée par Mouelle Mbassi, médecin albinos), mais également en Europe par deux femmes camerounaises – Adrienne Ntankeu en France (ANIDA, Association nationale, internationale pour la défense des albinos) et Annie Mokto en Belgique (Écran total). Cette dernière a écrit un livre poignant dans lequel elle retrace son enfance malheureuse au Cameroun, puis son épanouissement à l’âge adulte lorsqu'elle est arrivée en Belgique et où elle vit désormais [39]. Ensuite, de nombreux pays africains francophones (Gabon, Sénégal – ANAS, Association nationale des albinos du Sénégal –, Togo, Côte d'Ivoire, Burundi, RC et RDC) et anglophones (Malawi, Zambie – AIMZ, Albinos In Malawi and Zambia) ont créé leur association dirigée par des personnes albinos ou parents de personnes albinos. Souvent, la personnalité du Président fait bénéficier les Africains albinos d'une image positive, comme cela a été le cas pendant de nombreuses années pour Mwimba Texas, un catcheur bien connu à Kinshasa en RDC, malheureusement décédé en 2018, pour Thierno Diallo, albinos malien, qui a été ministre des Cultes pendant quelques années et pour Kazungu Kassim, albinos burundais, qui s'est consacré à la protection des personnes albinos dans son pays. Ce dernier a réussi à faire rassembler les enfants dans une école gardée par la police pour les protéger de toute attaque. Il est difficile de citer toutes ces associations, mais elles sont à présent regroupées au niveau mondial dans la GAA (Global Albinism Alliance, www.albinismalliance.org) dont la création doit beaucoup à Antoine Gliksohn de Genespoir (Association française des albinismes, www.genespoir.org).

C'est désormais la GAA qui organise tous les 2 ans une conférence internationale sur l'albinisme (ISCA, International Scientific Conference on Albinism, http://isca2022. albinismalliance.org). D'un point de vue historique, il faut mentionner la première association dans le monde fondée aux États-Unis en 1983 sous le nom de NOAH (National Organization for Albinism and Hypopigmentation, www.albinism.org) en raison de la fréquence importante de l'albinisme dans la communauté afro-américaine, mais aussi indienne et hispanique. C'est le Pr Carl J. Witkop, généticien à l'Université de Minneapolis, MN, l'un des premiers experts mondiaux de l'albinisme dans les années 1970, qui a écrit le premier article pour le N° 1 de la revue *NOAH* en 1983, intitulé « Noah – an albino » (« Noé – un albinos ») [51]. Ses successeurs, Richard King et William Oetting, ont fait entrer l'albinisme dans la génétique moléculaire [41]. N'oublions pas la fondation de Genespoir France à Rennes en 1995 par la mère d'un enfant albinos, à laquelle l'un d'entre nous (RA) a participé. Cette association est toujours en pleine activité et participe à de nombreux évènements scientifiques et relationnels (Fig. 1).

## Conclusion

Dans les pays d'Afrique subsaharienne, les personnes porteuses d'un albinisme oculocutané sont soumises dès leur naissance à une triple menace: une mauvaise vision qui va perturber leur éducation, l'absence de mélanine dans la peau qui va les classer parmi les « Africains blancs » sujets à de nombreux rituels généralement négatifs et surtout le développement de cancers cutanés très souvent mortels. Ce triptyque est à la base de notre plaidoyer en faveur de l'intégration de l'albinisme oculocutané parmi les Maladies tropicales négligées.

## Addendum

Le contenu de cet article a été partiellement présenté le 25 mai 2022 lors de la Journée scientifique de la SFMTSI consacrée aux « Maladies tropicales et pauvreté: impact sur les droits de la femme et de l'enfant ».

## Remerciements

Les auteurs remercient chaleureusement toutes les personnes albinos et non albinos qui depuis de nombreuses années nous ont apporté leur aide et nous ont fait partager leur expérience sur le terrain. Il est important de signaler le travail colossal réalisé par les associations de personnes albinos en France (Béatrice Jouanne, Genespoir) et en Afrique subsaharienne pour le plein épanouissement et la santé des personnes albinos: Jean Jacques Ndoudoumou, Luc Kamdem, Brice Temgoua, Raphael Kakmeni, Salomon Njike Kom, Madeleine Wafo, Caroline Nana, Adrienne Ntankeu et Annie Mokto au Cameroun, Mwinba Texas à Kinshasa et les frères Ndumba à Kisangani en RDC, Kassim Kasungu au Burundi, Kadidjatou Moumouni au Niger (ANAN, Association nationale des albinos du Niger), Salif Keita et Tierno Diallo au Mali, Rosalie Ngoua au Gabon, Sim Robinson au Togo, Bonface Massah au Malawi, Richard Nyathi au Zimbabwe. Toute notre reconnaissance à Madame Dominique Charmot pour son expertise dans l'organisation de la Figure 2.

## Contributions des auteurs

RA a pensé et rédigé le manuscrit, CB et PL ont apporté des contributions essentielles à la rédaction du manuscrit en raison de leur connaissance sur l'albinisme dans les pays anglophones africains. Tous les auteurs ont lu et approuvé le document final.

## Liens d'intérêts

Les auteurs ne déclarent aucun lien d'intérêts.
